# Sero-prevalence of *Helicobacter pylori* CagA immunoglobulin G antibody, serum pepsinogens and haemoglobin levels in adults

**DOI:** 10.1038/s41598-018-35937-9

**Published:** 2018-12-04

**Authors:** Khitam Muhsen, Ronit Sinnreich, Gany Beer-Davidson, Hisham Nassar, Daniel Cohen, Jeremy D. Kark

**Affiliations:** 1 Department of Epidemiology and Preventive Medicine, School of Public Health, Sackler Faculty of Medicine, Tel Aviv University Ramat Aviv, Tel Aviv, 6139001 Israel; 2 Hebrew University-Hadassah School of Public Health and Community Medicine, Jerusalem, Israel; 3 St. Joseph Hospital, East Jerusalem and Department of Cardiology, Hadassah-Hebrew University Medical Center, Ein Kerem, Jerusalem, 91120 Israel

## Abstract

Associations observed of *Helicobacter pylori* infection with haemoglobin levels are inconsistent. We examined associations of *H*. *pylori* sero-prevalence and serum pepsinogens (PGs), as non-invasive markers of atrophic gastritis, with haemoglobin levels. A cross-sectional study was undertaken among 654 Jewish and 937 Arab residents of Jerusalem, aged 25–78 years, randomly selected from Israel’s national population registry in age-sex and population strata. Sera were tested for *H*. *pylori* IgG, cytotoxin–associated gene A (CagA) antigen IgG antibody and PGs levels. Multivariable models were fitted to account for confounders. Participants with atrophic gastritis (PGI < 30 μg/L or a PGI: PGII < 3.0) had lower haemoglobin levels than those without: beta-coefficient −0.34 (95% CI −0.59, −0.09); in men −0.27 (95% CI −0.67, 0.12), and in women −0.43 (95% CI −0.74, −0.12). Lower haemoglobin levels were noted in persons with CagA antibody than in those *H*. *pylori* sero-negative or *H*. *pylor*i-CagA sero-negative: beta-coefficient −0.14 (95% CI −0.29, 0.01). Anaemia was more common among women and men with than without atrophic gastritis: adjusted OR 2.58 (95% CI 1.48, 4.48) and 1.52 (95% CI 0.59, 3.95), respectively. In conclusion, independent of known correlates, atrophic gastritis and apparently CagA sero-positivity, a marker of *H*. *pylori* virulent strains, are associated with lower haemoglobin levels.

## Introduction

Anaemia is an important public health problem, which usually results from a depletion of body iron stores. Prevalences of anaemia and iron deficiency anaemia (IDA) are increased by *Helicobacter pylori* infection (reviewed by Muhsen and Cohen^1^). *H*. *pylori*, a gram negative bacterium that colonizes the gastric mucosa and causes chronic gastritis^2^, is the main cause of gastric and duodenal ulcers, and an established risk factor for gastric cancer and MALT lymphoma^2–5^. These conditions usually develop in adulthood, although only in a subset of infected individuals^2^. In a recent meta-analysis we showed an increased likelihood of IDA in *H*. *pylori* infected persons vs uninfected ones: pooled odds ratio (OR) 1.72 (95% confidence interval (CI) 1.23–2.42)^6^. The association between *H*. *pylori* infection and all-cause anaemia was weaker: pooled OR 1.15 (95% CI 1.00–1.32)^6^. Currently, *H*. *pylori* eradication therapy is recommended in cases of refractory or unexplained IDA^7^.

Mechanisms that may explain associations of *H*. *pylori* infection with anaemia and IDA have not been fully elucidated, but pathogen-related factors might play a role. Systems of *H*. *pylori* iron regulation constantly express iron uptake, in contrast to systems of other bacteria^8^. *H*. *pylori* isolates from patients with IDA display a greater capability of iron uptake and of proliferation in the presence of iron compared to *H*. *pylori* isolates from non-IDA patients^9^. In addition, the expression of iron-repressible outer membrane proteins involved in iron acquisition differs between these groups^10^. Likely, some of these mechanisms enable survival of the bacteria in the hostile niche of the stomach, and also affect the host iron homeostasis. Evidence regarding the contribution of the cytotoxin-associated gene A (CagA) protein in the development of IDA remains inconclusive^10–15^, although this antigen was shown to be important in the pathogenesis of peptic disease and gastric cancer^2,5,16^. *H*. *pylori* chronic gastritis changes the physiology in the stomach^17^, including alterations in gastric acidity^17^ and ascorbic acid levels^18–20^, which are important in the absorption of dietary iron^21,22^.

Clearly, if associations of *H*. *pylori* infection with haemoglobin and other iron biomarkers is mediated by gastric inflammation, positive associations between *H*. *pylori*-related gastric pathology and anaemia are anticipated. However, the association of atrophic gastritis, a severe form of *H*. *pylori*-related gastric pathology, with haemoglobin, especially in the general population, was rarely addressed. *H*. *pylori* infection can cause atrophic body gastritis, which can lead to deficiencies in vitamin B12 and intrinsic factor, as well as hypochlorhydria; these negatively affect iron absorption^23,24^. Atrophic gastritis can be assessed using serum pepsinogen (PG) I and PGII, proenzymes of the digestive enzyme pepsin, which are secreted to the gastric lumen but which can also be detected in the serum^25–27^. With increasing severity of *H*. *pylori* gastritis, serum PGI and PGII levels are increased, but when atrophic changes occur in the corpus, PGI and the ratio of PGI: PGII decrease. More severe atrophy is related to a lower PGI: PGII ratio^25,28^.

The aims of the current study were to examine associations of *H*. *pylori* immunoglobulin G (IgG) sero-prevalence, CagA IgG sero-positivity and serum PGs, as non-invasive markers of atrophic gastritis, with two outcome variables: haemoglobin levels (continuous variable) and anaemia, in a population-based sample of adult men and women.

## Methods

### Study design and population

**Figure 1:**
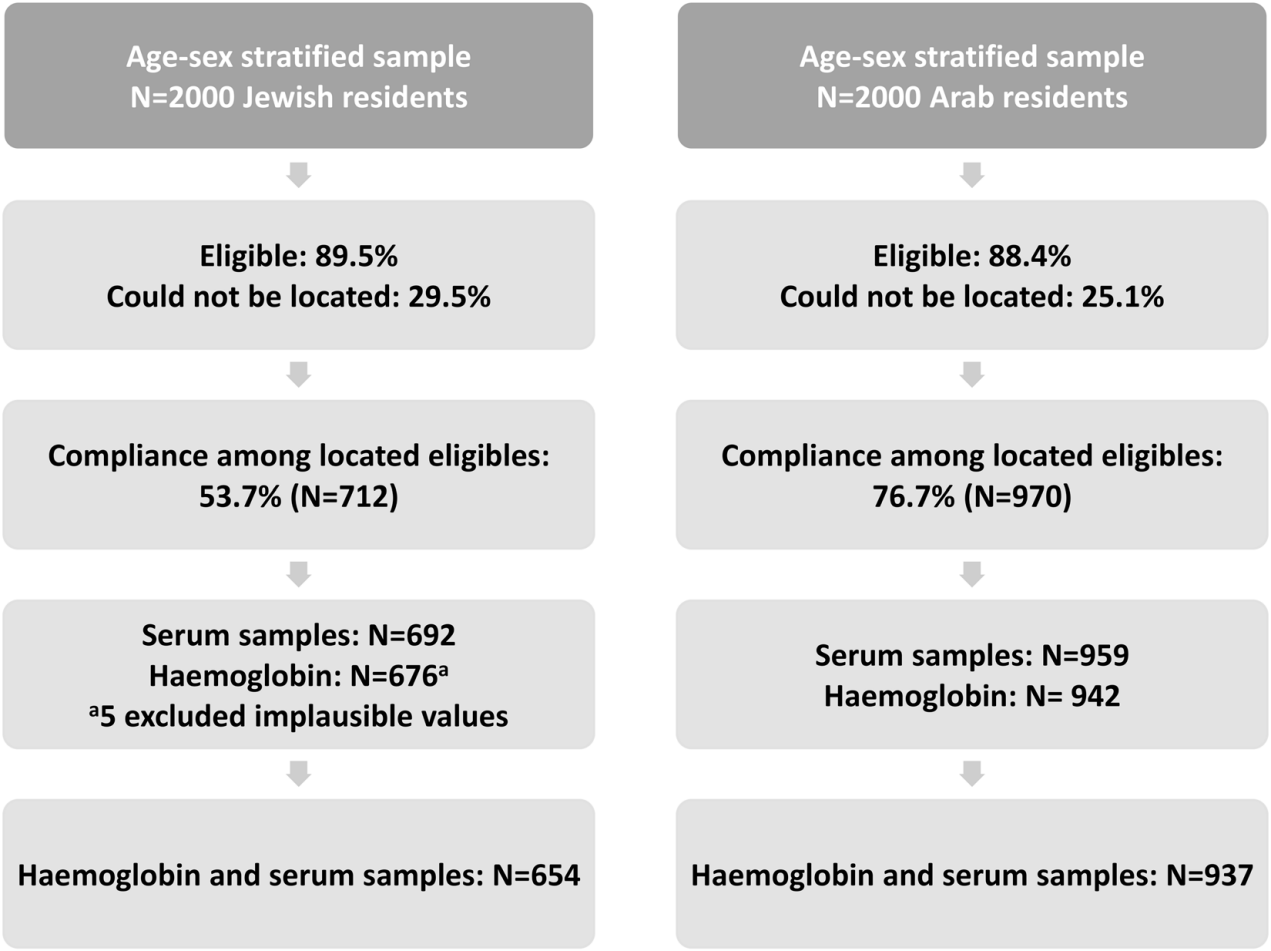
Sampling and enrolment.

We used archived anonymized (coded) serum samples obtained in the framework of a cross-sectional study of Jewish and Arab residents of Jerusalem. Details of the study design have been reported^29,30^. Briefly, age-sex-stratified random samples of 2000 Arab permanent residents of East Jerusalem and 2000 Israeli Jewish residents of Jerusalem, aged 25–74 years at sampling, were drawn from the Israel national population registry. Individuals were ineligible if they were institutionalized, housebound, had a severe illness, were unable to provide informed consent, pregnant or had given birth within three months preceding study initiation. The response rates among those located were 76.7% for Arabs (N = 970) and 53.7% for Jews (N = 712)^29,30^. For the current study, data were available for 937 (96.6%) and 654 (91.9%) Arab and Jewish participants, respectively (Fig. 1). Data were collected by personal interviews. Information was obtained on age at enrolment (grouped as 25–44, 45–64, and 65–78 years), sex and education (classified as having an academic degree/education, high-school/some college, some high school or less). Regular smoking was defined as reported smoking of at least one cigarette daily. Weight and height were measured. Body mass index (BMI) was calculated using measured weight and height as: weight (in kilograms [kg]) divided by height^2^ (in meters [m]); obesity was defined as BMI ≥30 kg/m^2^. Blood samples were collected after a 12 hr fast, and haemoglobin levels were measured by auto-analyser. Haemoglobin levels lower than 12 g/dL in women and lower than 13 g/dL in men were employed to define anaemia. Anaemia was classified as microcytic, normocytic and macrocytic if values of mean corpuscular volume (MCV) were <80 fL, 80–100 fL and >100 fL, respectively.

### Laboratory methods

Sera were tested for the presence of specific *H*. *pylori* IgG antibodies using enzyme-linked immunosorbent assay (ELISA) (Enzygnost^®^ Anti-*Helicobacter pylori* II/IgG kit, Siemens Diagnostics Product GmbH, Marburg, Germany). Optical density values >0.250 were classified as *H*. *pylori* sero-positive following the manufacturer’s instructions. Sensitivity and specificity values of the kit are within the range of 94–98%. The detection of *H*. *pylori* IgG serum antibody using this kit was significantly correlated with the detection of *H*. *pylori* antigen in stool samples that used monoclonal antigen detection enzyme immunoassay (Spearman’s coefficient 0.70, P < 0.001) (Muhsen *et al*., unpublished). The presence of IgG antibody against recombinant CagA protein^31^ was measured in *H*. *pylori-*positive sera, employing a modified in-house ELISA protocol, as previously described^31^. The detection of CagA IgG serum antibody with this recombinant CagA protein has demonstrated high sensitivity (>90%) in identifying CagA strains^32^. Participants were classified as: a) *H*. *pylori* seronegative; b) *H*. *pylori* positive, CagA negative, if they had *H*. *pylori* IgG antibodies, but lacked CagA IgG antibodies; or c) *H*. *pylori* positive, CagA positive if they were positive for *H*. *pylori* and CagA IgG antibodies.

Concentrations of serum PGI and PGII were quantified by ELISA (Biohit Inc., Helsinki, Finland). Atrophic gastritis was defined as serum PGI levels of <30 μg/L or a PGI: PGII ratio of <3.0, as recommended by the manufacturer. Higher serum PGI and PGII levels are found in *H*. *pylori* infected vs uninfected individuals, while a lower PGI: PGII ratio is found in the former^28^. In our sample, *H*. *pylori* sero-status was significantly correlated with PGI level (Spearman’s coefficient 0.19, P < 0.001), PGII level (Spearman’s coefficient 0.33, P < 0.001) and PGI: PGII ratio (Spearman’s coefficient −0.23, P < 0.001). These correlations strengthened the validity of the classification of *H*. *pylori* sero-status.

### Statistical analysis

Student’s *t* tests and one-way analysis of variance (ANOVA) were used to examine unadjusted differences in mean haemoglobin levels according to sociodemographic variables, *H*. *pylori* sero-status, atrophic gastritis and smoking. When more than two strata of a variable were compared, we used a Tukey test to account for multiple comparisons. Multivariable linear regression models with haemoglobin level as the dependent variable were fitted; from these models, we obtained beta (slope) coefficients (and 95% CIs). Categorical independent variables were included in the model as dummy variables. Chi square tests were employed to examine unadjusted associations of sociodemographic variables, *H*. *pylori*-CagA IgG antibody sero-status and atrophic gastritis, with anaemia. Multivariable logistic regression models were fitted, from which we obtained adjusted ORs and 95% CIs. Variables associated with the dependent variable (haemoglobin in linear regression and anaemia in logistic regression) in bivariate analysis with P < 0.2 were included in the multivariable analysis, in addition to age, atrophic gastritis (as measured by serum PGs) and *H*. *pylori* infection. As haemoglobin levels differ between men and women, the analyses were conducted in sex-specific strata. Interactions between population group, age, sex, *H*. *pylori* sero-status and atrophic gastritis were assessed. Data were analysed using IBM SPSS (Armonk, New York, USA) version 23 and Winpepi^33^.

### Ethics statement

The study was approved by the Institutional Review Board of the Hadassah Medical Centre, Jerusalem, and by the ethics committee at Tel Aviv University. All participants signed an informed consent. The study was conducted in accordance with the Declaration of Helsinki ethical principles and regulation of the Ministry of Health.

## Results

Information on haemoglobin and *H*. *pylori* sero-status was available for 937 and 654 Arab and Jewish participants, respectively; of these 498 (53.1%) and 348 (53.2%) were men (P = 0.9), respectively. The mean ages were 52.0 (SD 13.9) and 52.4 (13.6) years (P = 0.5) for Jews and Arabs, respectively. The mean haemoglobin level was higher among men than among women: 15.0 g/dL (SD 1.5) vs. 12.9 g/dL (SD 1.3), P < 0.001. Anaemia was evident in 206 participants: 12.9% (95% CI 11.4–14.7%). Among these, 64 (31.0%) had microcytic anaemia, 139 (67.5%) had normal MCV values and three (1.5%) had macrocytic anaemia. Anaemia was more prevalent among women (20.3% [95% CI 17.5%, 23.3%]) than men (6.5% [95% CI 5.0%, 8.3%]), P < 0.001.

### Haemoglobin levels by demographic variables, *H. pylori* IgG sero-positivity and atrophic gastritis in men

The mean haemoglobin level decreased with age (P < 0.001). Differences between the age groups in mean haemoglobin level remained statistically significant after correction for multiple comparisons (by Tukey). A higher mean haemoglobin level was found in smokers than in non-smokers (P < 0.001). No significant difference was found in mean haemoglobin level according to education and according to *H*. *pylori* sero-status. Men with evidence of atrophic gastritis had a lower mean haemoglobin level than those without (P = 0.037) (Table 1).

**Table 1:** Unadjusted mean haemoglobin levels according to demographic and behavioural variables, *H*. *pylori* sero-prevalence and atrophic gastritis by sex*.

	N	Men	P	N	Women	P
		Mean haemoglobin (SD)			Mean haemoglobin (SD)	
**Population**			0.11			<0.001
Jews	348	15.1 (1.5)		306	13.3 (1.2)	
Arabs	498	15.0 (1.4)		439	12.7 (1.3)	
**Age** (**years**)			<0.001^†^			0.005^†^
25–44	297	15.4 (1.4)		234	12.7 (1.2)	
45–64	358	15.1 (1.3)		337	13.0 (1.3)	
65–78	191	14.4 (1.5)		174	13.0 (1.4)	
**Education**			0.2^†^			0.013^†^
Some high school or less	430	15.1 (1.5)		418	12.8 (1.3)	
High school certificate/some college	200	14.9 (1.5)		166	12.9 (1.3)	
Academic education	211	15.1 (1.4)		160	13.2 (1.2)	
**Smoking**			<0.001			0.03
Regular smoking ≥1 cigarette/ day	290	15.4 (1.3)		75	13.2 (1.4)	
Other	550	14.9 (1.5)		663	12.9 (1.3)	
**Obesity**			0.9			0.3
BMI <30 kg/m^2^	615	15.0 (1.5)		404	13.0 (1.3)	
BMI ≥30 kg/m^2^	228	15.0 (1.4)		341	12.9 (1.2)	
***H***. ***pylori*** **IgG sero-status**			0.6^†^			0.012^†^
Negative	217	15.0 (1.6)		189	12.9 (1.3)	
*H*. *pylori* positive CagA negative	388	15.1 (1.5)		333	13.0 (1.2)	
*H*. *pylori* positive CagA positive	241	15.0 (1.3)		223	12.7 (1.4)	
**Atrophic gastritis**: PGI <30 μg/L or PGI: PGII <3.0			0.037			0.052
No	790	15.0 (1.4)		671	13.0 (1.2)	
yes	52	14.6 (1.7)		71	12.5 (1.7)	

^*^BMI: body mass index, CagA: cytotoxin associated gene A; kg: kilogram, m: meters, PG: pepsinogen, SD: standard deviation.

^†^P value for the difference between the groups by one-way analysis of variance (ANOVA).

**Pair comparisons by Tukey HSD Post-hoc Test – Men**:

Age group: 25–44 vs 45–64 years P = 0.03; age group 24–44 vs 65–78 years P < 0.001; age group 45–64 vs 65–78 years P < 0.001.

Education: Some high school or less vs high school certificate/some college P = 0.37; some high school or less vs academic education P = 0.86, high school certificate/some college vs academic education P = 0.27. *H*. *pylori* sero-status: Negative vs *H*. *pylori* positive CagA negative P = 0.7; negative vs *H*. *pylori* positive CagA positive P = 0.9; *H*. *pylori* positive CagA negative vs *H*. *pylori* positive CagA positive P = 0.6.

**Pair comparisons by Tukey HSD Post-hoc Test – Women**:

Age group: 25–44 vs 45–64 years P = 0.004; age group 24–44 vs 65–78 years P = 0.026; age group 45–64 vs 65–78 years P = 0.9.

Education: Some high school or less vs high school certificate/some college P = 0.6; some high school or less vs academic education P = 0.011, some high school or less vs academic education P = 0.2. *H*. *pylori* sero-status: Negative vs *H*. *pylori* positive CagA negative P = 0.5; negative vs *H*. *pylori* positive CagA positive P = 0.2; *H*. *pylori* positive CagA negative vs *H*. *pylori* positive CagA positive P = 0.009.

On multivariable analysis, men with evidence of atrophic gastritis had non-significantly lower haemoglobin levels: beta coefficient −0.27 (95% CI −0.67, 0.12), P = 0.17 (Table 2). No statistically significant difference was found in haemoglobin levels according to CagA IgG sero-status. The associations of age and smoking with haemoglobin level persisted in this model, which also showed lower haemoglobin levels in Arab than Jewish men (Table 2). No interactions were detected between population group and atrophic gastritis (P = 0.7), population group and CagA IgG antibody sero-status (P = 0.9), and CagA IgG antibody sero-status and atrophic gastritis (P = 0.7).

**Table 2:** Multiple linear regression analysis of haemoglobin levels according to demographic and behavioural variables, *H*. *pylori* sero-prevalence and serological evidence of atrophic gastritis*.

Variable	Pooled sexes**	P	Men**	P	Women**	P
	Beta coefficient (95% CI)		Beta coefficient (95% CI)		Beta coefficient (95% CI)	
Sex (Males vs females)	1.98 (1.84, 2.12)	<0.001	—		—	
Age 24–44 years	Reference		Reference		Reference	
Age 45–64 years	0.02 (−0.13, 0.18)	0.7	−0.23 (−0.45, −0.01)	0.04	0.34 (0.13, 0.56)	0.002
Age 65–78 years	−0.29 (−0.48, −0.10)	0.002	−0.85 (−1.12, −0.59)	<0.001	0.37 (0.11, 0.63)	0.006
Population group (Arabs vs Jews)	−0.36 (−0.59, −0.09)	<0.001	−0.20 (−0.40, 0.00)	0.05	−0.48 (−0.69, −0.27)	<0.001
Regular smoking ≥1 cigarette/ day (reference: other)	0.48 (0.31, 0.65)	<0.001	0.45 (0.24, 0.66)	<0.001	0.29 (−0.005, 0.60)	0.054
Atrophic gastritis (PGI <30 µg/L or PGI: PGII ratio <3) (yes vs no)	−0.34 (−0.59, −0.09)	0.009	−0.27 (−0.67, 0.12)	0.17	−0.43 (−0.74, −0.12)	0.007
*H*. *pylori* positive CagA positive (yes vs no)	−0.14 (−0.29, 0.01)	0.069	−0.09 (−0.29, 0.13)	0.4	−0.15 (−0.35, 0.06)	0.15
Education: Some high school or less	−0.05 (−0.29, 0.13)	0.5	−0.02 (−0.26, 0.23)	0.8	−0.11 (−0.37, 0.15)	0.4
High school certificate/some college	−0.16 (−0.36, 0.04)	0.12	−0.23 (−0.51, 0.04)	0.099	−0.04 (−0.32, 0.25)	0.8
Academic education	Reference		Reference		Reference	

^*^CagA: cytotoxin associated gene A; CI: confidence intervals; PG: pepsinogen.

^**^Adjusted for the variables in the table. R Square 0.4 for the pooled model, 0.09 for men and 0.08 for women.

### Haemoglobin levels by demographic variables, *H*. *pylori* IgG sero-positivity and atrophic gastritis in women

Lower mean haemoglobin levels were found among Arab than Jewish women. A gradient was observed in relation to education (Table 1); namely, women with some high school education or less had significantly lower haemoglobin levels than did those with an academic degree (P = 0.011 by Tukey test). The average haemoglobin level was lower in women aged 25–44 years than in women aged 45–64 years (P = 0.004 by Tukey test), and than in women aged 65–78 years (P = 0.026 by Tukey test); and it was higher in smokers than in non-smokers (P = 0.03). Women with *H*. *pylori* CagA serum IgG antibody had a lower mean haemoglobin level than *H*. *pylori* sero-positive women who were lacking serum CagA IgG antibody (P = 0.009 by Tukey test), but the level was similar to that of *H*. *pylori* negative women. Women with serological evidence of atrophic gastritis had a lower mean haemoglobin level than women without (P = 0.052) (Table 1). The differences in haemoglobin levels according to atrophic gastritis persisted in a multivariable analysis (P = 0.007); the association with CagA IgG sero-positivity did not (P = 0.15) (Table 2). No significant interaction was found between population group and atrophic gastritis (P = 0.2), population group and CagA IgG antibody sero-status (P = 0.6), and CagA sero-status and atrophic gastritis (P = 0.10).

In a pooled multivariable analysis of both sexes, men had a higher mean haemoglobin level than women. Participants with atrophic gastritis had significantly lower haemoglobin levels than those without (P = 0.009). CagA IgG sero-positivity was related to a lower mean haemoglobin level (P = 0.069) (Table 2). No significant interactions were found between sex and atrophic gastritis (P = 0.8) or by CagA IgG sero-positivity (P = 0.3).

### Variables associated with anaemia

Among men, a non-significantly higher prevalence of anaemia was found among those with evidence of atrophic gastritis than those without (P = 0.13). Age was positively related to anaemia prevalence, while a lower prevalence of anaemia was found in smokers than non-smokers (P < 0.001). No difference was noted in the prevalence of anaemia according to *H*. *pylori* IgG sero-prevalence (Table 3). The results were similar in a multivariable logistic regression model that adjusted for age and *H*. *pylori* sero-positivity (Table 4). Among women, the prevalence of anaemia was higher among those who had atrophic gastritis than those without (P = 0.001) (Table 3). Anaemia prevalence was higher among women with CagA IgG serum antibody than among those who were *H*. *pylori* sero-negative, and those who lacked CagA IgG antibodies. The prevalence of anaemia was higher among Arab than Jewish women. These associations persisted in a multivariable analysis that included age and *H*. *pylori* CagA sero-positivity in addition to population group, education and smoking (Table 4).

**Table 3:** Associations of sociodemographic and behavioural variables, *H*. *pylori* sero-prevalence and serological evidence of atrophic gastritis with anaemia by sex.

	Men	P*	Women	P*
	Anaemia/total (%)		Anaemia/total (%)	
**Population group**		0.11		<0.001
Jews	17/348 (4.9)		36/306 (11.8)	
Arabs	38/498 (7.6)		115/439 (26.2)	
**Age**, **years**		<0.001		0.062
25–44	10/297 (3.4)		59/234 (25.2)	
45–64	16/358 (4.6)		58/337 (17.2)	
65–78	29/191 (15.2)		34/174 (19.5)	
**Education**		0.17		0.021
Some high school or less	22/430 (5.1)		98/418 (23.4)	
High school certificate/some college	18/200 (9.0)		32/166 (19.3)	
Academic education	13/211 (6.2)		21/160 (13.1)	
**Smoking**		<0.001		0.4
Regular smoking ≥1 cigarette/ day	6/291 (2.1)		13/75 (17.3)	
Other	49/550 (8.9)		137/663 (20.7)	
**Obesity**		0.9		0.8
BMI <30 kg/m^2^	40/615 (6.5)		81/404 (20.0)	
BMI ≥30 kg/m^2^	15/228 (6.6)		70/341 (20.5)	
***H***. ***pylori*** **IgG sero-status**		0.6		0.037
Negative	17/216 (7.8)		35/189 (18.5)	
*H*. *pylori* positive CagA negative	24/338 (6.2)		58/333 (17.4)	
*H*. *pylori* positive CagA positive	14/241(5.8)		58/223 (26.0)	
**Atrophic gastritis** (PGI <30 μg/L, and/or a PGI: PGII <3.0)		0.13		0.001
No	49/789 (6.2)		125/671 (18.6)	
yes	6/52 (11.5)		25/71 (35.2)	

^*^P values were obtained by chi square test.

BMI: body mass index, CagA: cytotoxin associated gene A; kg: kilogram, m: meters, PG: pepsinogen, SD: standard deviation.

**Table 4:** Multivariable logistic modelling of determinants of anaemia, stratified by sex*.

	Adjusted OR (95% CI)*	P
Men**		
Age, years		<0.001 (2df)
25–44	Reference	
45–64	1.21 (0.53, 2.81)	0.6
65–78	4.63 (2.12, 10.09)	<0.001
Population group: Arabs vs Jews	2.17 (1.13, 4.16)	0.019
Atrophic gastritis (PGI <30 µg/L or PGI: PGII ratio <3) (yes vs no)	1.52 (0.59, 3.95)	0.3
*H*. *pylori* IgG positive CagA IgG positive vs the rest	0.89 (0.46, 1.72)	0.7
Regular smoking ≥1 cigarette/ day vs other	0.20 (0.08, 0.53)	0.001
Education:		0.11 (2df)
Some high school or less	0.67 (0.31,1.45)	0.3
High school certificate/some college	1.38 (0.63–3.04)	0.4
Academic education	Reference	
Women**		
Age, years		0.021 (2df)
25–44	Reference	
45–64	0.55 (0.36, 0.85)	0.007
65–78	0.60 (0.36, 1.01)	0.056
Population group: Arabs vs Jews	2.15 (1.36, 3.38)	0.034
Atrophic gastritis (PGI <30 µg/L or PGI: PGII ratio <3) (yes vs no)	2.58 (1.48, 4.48)	0.001
*H*. *pylori* IgG positive CagA IgG positive vs the rest	1.40 (0.94, 2.07)	0.09
Regular smoking ≥1 cigarette/ day vs other	0.89 (0.47, 1.69)	0.7
Education:		0.5 (2df)
Some high school or less	1.31 (0.74, 2.34)	0.3
High school certificate/some college	1.06 (0.56, 1.99)	0.8
Academic education	Reference	

*CagA: cytotoxin associated gene A; CI: confidence intervals; df: degrees of freedom; OR: odd ratio; PG: pepsinogen.

Adjusted for the variables included in the table.

**For men Nagelkerke R Square = 0.157, P = 0.9 by Hosmer and Lemeshow Test.

**For women Nagelkerke R Square = 0.091, P = 0.48 by Hosmer and Lemeshow Test.

In a secondary analysis, we re-grouped the study participants according to values of the PGI: PGII ratio that correlated with gastritis severity, using the OLGA system^34^: most severe <3.0, moderate 3.0–6.8 and least severe >6.8. With these cut-off values, the mean haemoglobin levels were 14.6 (SD 1.6), 15.0 (SD 1.4) and 15.0 (SD 1.4), respectively, in men (P = 0.2 by ANOVA). In women, the corresponding values were 12.6 (SD 1.6), 12.7 (SD 1.3) and 13.0 (SD 1.2) (P = 0.029 by ANOVA). In a multivariable analysis, the significant gradient between PGI: PGII and haemoglobin level was maintained in women (Supplementary Table S1).

### Anaemia sub-type according to *H*. *pylori* sero-status and atrophic gastritis

Overall, the prevalence of microcytic anaemia was 5.2%, 3.1% and 4.5% in persons who were *H*. *pylori* sero-negative, *H*. *pylori* sero-positive but lacking CagA IgG antibody, and *H*. *pylori* sero-positive CagA positive, respectively. The respective prevalences for normocytic anaemia were 7.6%, 8.3% and 10.3%. The prevalence of microcytic anaemia was 9.8% in persons with atrophic gastritis vs 3.6% in those without this condition. The respective prevalence for normocytic anaemia was 8.1% vs 15.4%. All three persons with macrocytic anaemia were positive for *H*. *pylori* CagA IgG but without atrophic gastritis (Supplementary Table S2).

## Discussion

We examined associations of *H*. *pylori* IgG sero-prevalence, CagA IgG sero-positivity and serum PGs, as non-invasive markers of atrophic gastritis, with haemoglobin levels and anaemia, in men and women of two ethnic groups in a general population.

Serologic evidence of atrophic gastritis was associated with a higher prevalence of anaemia, particularly in women, and with lower mean haemoglobin levels (mean difference 0.34 g/dL). A similar trend, although of smaller magnitude, was found in relation to CagA IgG antibody sero-positivity (difference of 0.14 g/dL in mean haemoglobin level). These differences were more evident in women, although no significant interaction was found between sex and atrophic gastritis or between sex and CagA IgG sero-positivity. Notably, these observations were independent of age, population group, education and smoking history. A limited number of studies have addressed associations of *H*. *pylori* infection with anaemia or haemoglobin level among adults in the general population^35–40^. These mostly showed no significant difference between infected and uninfected individuals in mean haemoglobin levels or in the prevalence of anaemia, except for studies carried out in pregnant women^39,40^. None of these studies has addressed the role of CagA infection or atrophic gastritis. Our findings add a new dimension, suggesting that severe gastric inflammation, even with atrophic gastritis (as evident by serum PGs levels), rather than exposure to *H*. *pylori* per se, are involved in decreased haemoglobin levels. Thus, our results improve the risk profiling of low haemoglobin levels in relation to *H*. *pylori* infection. In dyspeptic adult patients, lower mean haemoglobin levels and a higher prevalence of anaemia were documented in *H*. *pylori* infected compared to uninfected patients^41^. A case-control study from the United Kingdom^42^ showed that adult patients referred for investigation of IDA had significantly more frequent gastric body atrophy, as demonstrated by gastric biopsy, compared to control patients with normal haemoglobin and iron levels. A gradient was observed with increased atrophy grades, whereas *H*. *pylori* infection as a main effect was not associated with IDA^42^. Anaemic patients with gastric body atrophy were less likely to have conditions that might be the definite cause of anaemia than were anaemic patients without atrophy. This suggests that gastric body atrophy might be a cause of anaemia in some individuals and a contributory factor in others^42^. Nahon el al.^43^ showed that patients with unexplained IDA referred for gastric tract evaluation had a higher prevalence of chronic gastritis than control patients (67% vs 47%), and than patients with atrophic gastritis (15% vs 6%) and *H*. *pylori* infection (60% vs 43%)^43^. Similar to our observations, Lee and colleagues^44^ showed in a general population sample of 2398 participants, lower haemoglobin levels and higher anaemia prevalence among persons with serological evidence of gastric atrophy (PGI: PGII ratio < 3.0, either with or without *H*. *pylori* serum antibodies) compared to those without gastric atrophy^44^. Collectively, these and our findings confirm the hypothesis that the association of *H*. *pylori* infection with lower haemoglobin might be mediated by *H*. *pylori*-associated inflammatory and atrophic changes affecting the gastric mucosa. Anaemia is an alarm symptom of gastric cancer that prompts invasive diagnostic techniques. Atrophic gastritis is mostly caused by *H*. *pylori* infection, and is a main precursor of gastric cancer^45^. A positive association between anaemia and serologic evidence of atrophic gastritis suggests that anaemia might be a marker of pre-malignant lesions in the stomach. This observation may promote early detection of gastric cancer.

Atrophic lesions in the stomach affect gastric acidity^17^ and ascorbic acid levels^18–20^, which are essential to the absorption of dietary iron^21,22^. Body gastric atrophy is also associated with decreases in secretion of intrinsic factor and in absorption of dietary vitamin B12, which may result in pernicious anaemia^23,24^. CagA is associated with an inflammatory response in the stomach, but also has the potential to induce carcinogenesis irrespective of inflammation (reviewed in^31^). We found a clear trend of higher prevalence of microcytic anaemia in persons with serological evidence of atrophic gastritis compared to persons without such condition, and all three persons with macrocytic anaemia tested positive for CagA IgG serum antibody.

Consistent with previous reports^46–48^, women aged 25–44 years had lower haemoglobin levels than did older women, thus conferring a risk for anaemia among women of child-bearing age; this is likely due to menstrual blood loss and enhanced demands related to pregnancy. In men, however, haemoglobin levels decreased with advanced age. A longitudinal study from Japan demonstrated that haemoglobin levels declined as age increased in men, while in women a decline was observed after the age of 60 years^49^. Age-related patterns in haemoglobin levels likely reflect the ageing process, but might also be related to a birth cohort effect, or both, as has been shown^49^.

Our results also confirmed previous observations of higher haemoglobin levels among smokers than non-smokers^46,47,49,50^. This finding apparently results from compensatory erythropoiesis stimulated by smoking-induced hypoxia.

Lower haemoglobin levels were found among Arab than Jewish patients, and Arabs tended to be more anaemic. These differences might reflect differences in socioeconomic status and dietary habits. Genetic differences may also contribute to the Arab-Jewish gap in anaemia prevalence. Consanguineous marriages are far more common in the Arab population in Israel^51^, as are haemoglobinopathies^52^.

Our study has limitations. The cross-sectional design limits causal inference regarding associations of *H*. *pylori* infection and atrophic gastritis with anaemia. Nonetheless, *H*. *pylori* infection is typically acquired in early childhood and, without treatment, persists for life. Hence, the *H*. *pylori* infection likely preceded the occurrence of anaemia, which presumably developed in adulthood. *H*. *pylori* is a main cause of atrophic gastritis, and when the latter ensues, the bacterium usually loses its niche and disappears. Hence, it is likely that some persons who were classified as *H*. *pylori* sero-negatives were actually previously infected with the bacterium. Such a scenario would likely result in underestimation of associations of *H*. *pylori* infection with haemoglobin levels and anaemia. We cannot determine whether atrophic gastritis resulted from *H*. *pylori* infection or autoimmunity. Addressing such a question is especially challenging given the overlap between these two conditions^53^ and the evidence showing that *H*. *pylori* might play a role in gastric autoimmunity via molecular mimicry^23,54^.

Our use of serum PGs as a surrogate marker to define atrophic gastritis might have limited sensitivity. However, we examined in the serum, both PGI concentration and PGI: PGII ratio; a combination of these parameters has been shown to improve the accuracy of detection. A PGI level of 25–30 µg/L or less and PGI: PGII < 3.0 are commonly used cut-off values^55,56^ (reviewed by Zagari *et al*.^26^) when using Biohit ELISAs, with sensitivity ranging between 71% and 90%, and high specificity of 90–98% compared to gastric biopsy^56–58^; this usually detects moderate to severe forms of atrophic gastritis.

Our study has a number of strengths. First, it comprises a large sample size of men and women from two general population samples. Second, persons with conditions that might induce anaemia, such as cancer, severe kidney disease, pregnancy and recent birth were excluded from the study. Third, we were able to adjust for potential confounders, which were not available in many of the previous studies that assessed the association between *H*. *pylori* infection and anaemia.

In summary, over and above known correlates of haemoglobin levels and anaemia, we found that serological evidence of atrophic gastritis was associated with lower mean haemoglobin levels, mainly in women, and a similar trend, although of smaller magnitude, was found in relation to CagA IgG antibody sero-positivity. Our results provide new insight regarding populations at risk for low haemoglobin levels in relation to *H*. *pylori* infection and its related gastritis, as measured non-invasively by serum pepsinogen levels.

## Electronic supplementary material

Supplementary tables

## Data Availability

Data can be provided upon request to the corresponding author (KM).
