# Supplementary material for: Sero-prevalence of Helicobacter pylori CagA immunoglobulin G antibody, serum pepsinogens and haemoglobin levels in adults
Source: Sci Rep. 2018 Dec 4;8:17616. doi: 10.1038/s41598-018-35937-9 (PMC6279832; doi:10.1038/s41598-018-35937-9)
Supplement: Supplementary file 1 — Supplementary tables [file 41598_2018_35937_MOESM1_ESM.pdf]

Sero-prevalence of *Helicobacter pylori* CagA immunoglobulin G antibody, serum pepsinogens and haemoglobin levels in adults

Khitam Muhsen[1*], Ronit Sinnreich [2], Gany Beer-Davidson[1], Hisham Nassar[3], Daniel Cohen[1+], Jeremy D. Kark[2, 4+]

[+]These authors contributed equally as senior authors

[1] Department of Epidemiology and Preventive Medicine, School of Public Health, Sackler Faculty of Medicine, Tel Aviv University Ramat Aviv, Tel Aviv, Israel

[2] Hebrew University-Hadassah School of Public Health and Community Medicine, Jerusalem, Israel

[3]St. Joseph Hospital, East Jerusalem and Department of Cardiology, Hadassah-Hebrew University Medical Center, Ein Kerem, Jerusalem 91120, Israel

[4] Deceased

Supplementary Table S1: Multiple linear regression analysis of haemoglobin levels according to demographic and behavioural variables, *H. pylori* sero-prevalence and serological evidence of atrophic gastritis *

	Pooled sexes**		Men**		Women**	
Variable	Beta coefficient (95% CI)	P	Beta coefficient (95% CI)	P	Beta coefficient (95% CI)	P
Sex (Males vs females)	1.99 (1.85, 2.14)	<0.001	-		-	
Age 24-44 years	Reference		Reference		Reference	
Age 45-64 years	0.001 (-0.16, 0.16)	0.9	-0.28 (-0.49, -0.05)	0.015	0.33 (0.11, 0.54)	0.003
Age 65-78 years	-0.32 (-0.51, -0.13)	0.001	-0.92 (-1.19, -0.65)	<0.001	0.35 (0.09, 0.61)	0.009
Population group (Arabs vs Jews)	-0.36 (-0.51, -0.22)	<0.001	-0.22 (-0.402, 0.01)	0.036	-0.48 (-0.69, -0.27)	<0.001
Regular smoking ≥1 cigarette/ day (reference: other)	0.46 (0.29, 0.63)	<0.001	0.45 (0.24, 0.66)	<0.001	0.26 (-0.04, 0.57)	0.089
**PGI: PGII *****						
<3.0	-0.27 (-0.53, -0.001)	0.049	-0.12 (-0.53, 0.29)	0.5	-0.44 (-0.77, -0.10)	0.011
3.0-6.8	-0.02 (-0.17, 0.12)	0.7	0.18 (-0.03, 0.39)	0.095	-0.19 (-0.39, 0.002)	0.052
>6.8	Reference		Reference		Reference	
H. pylori positive CagA positive (yes vs no)	-0.14 (-0.29, 0.01)	0.072	-0.13 (-0.35, 0.90)	0.2	-0.12 (-0.33, 0.08)	0.2
Education: Some high school or less	-0.05 (-0.23, 0.13)	0.5	-0.03 (-0.28, 0.22)	0.8	-0.10 (-0.36, 0.16)	0.4
High school certificate/some college	-0.18 (-0.38, 0.02)	0.081	-0.24 (-0.52, 0.04)	0.09	-0.05 (-0.34, 0.23)	0.7
Academic education	Reference		Reference		Reference	

* CagA: cytotoxin associated gene A, CI: confidence intervals; PG: pepsinogen
** Adjusted for the variables in the table. R Square 0.399 for the pooled model, 0.085 for men and 0.063 for women. *** These values were shown to be related to the severity of gastritis using the Operative Line for Gastritis Assessment (OLGA) staging[34].

Supplementary Table S2: Prevalence of anaemia subtype by *H. pylori* IgG sero-status and serological evidence of atrophic gastritis

	Total	Microcytic anaemia	Normocytic anaemia	Macrocytic anaemia	P
***H. pylori* IgG sero-status**					0.039 (df=6)
Negative	406	21 (5.2%)	31 (7.6%)	0 (0%)	
H. pylori positive CagA negative	721	22 (3.1%)	60 (8.3%)	0 (0%)	
H. pylori positive CagA positive	464	21 (4.5%)	48 (10.3%)	3 (0.6%)	
**Atrophic gastritis** (PGI <30 μg/L, or a PGI: PGII <3.0)					<0.001
No	1461	52 (3.6%)	119 (8.1%)	3 (0.2%)	
Yes	123	12 (9.8%)	19 (15.4%)	0 (0%)	

CagA: cytotoxin associated gene A; PG: pepsinogen

P value was obtained by chi square test

Anaemia was defined as haemoglobin levels lower than 12 g/dL in women and lower than 13g/dL in men. Anaemia was classified as microcytic, normocytic and macrocytic if values of mean corpuscular volume were lower than 80 fL, 80-100 fL and greater than 100 fL, respectively.
